# DNA methylation landscape of fat deposits and fatty acid composition in obese and lean pigs

**DOI:** 10.1038/srep35063

**Published:** 2016-10-10

**Authors:** Shunhua Zhang, Linyuan Shen, Yudong Xia, Qiong Yang, Xuewei Li, Guoqing Tang, Yanzhi Jiang, Jinyong Wang, Mingzhou Li, Li Zhu

**Affiliations:** 1 College of Animal Science and Technology, Sichuan Agricultural University, Chengdu, Sichuan, China; 2 E-GENE, Shenzhen, Guangdong, China; 3 Department of Animal Husbandry and Veterinary Medicine, Chengdu Agricultural College, Chengdu, Sichuan, China; 4 Department of Biology, College of Life and Science, Sichuan Agricultural University, Chengdu, Sichuan, China; 5 Chongqing Academy of Animal Science, Rongchang, Chongqing, China

## Abstract

Obese and lean type pig breeds exhibit differences in their fat deposits and fatty acid composition. Here, we compared the effect of genome-wide DNA methylation on fatty acid metabolism between Landrace pigs (LP, leaner) and Rongchang pigs (RP, fatty). We found that LP backfat (LBF) had a higher polyunsaturated fatty acid content but a lower adipocyte volume than RP backfat (RBF). LBF exhibited higher global DNA methylation levels at the genome level than RBF. A total of 483 differentially methylated regions (DMRs) were located in promoter regions, mainly affecting olfactory and sensory activity and lipid metabolism. In LBF, the promoters of genes related to ATPase activity had significantly stronger methylation. This fact may suggest lower energy metabolism levels, which may result in less efficient lipid synthesis in LBF. Furthermore, we identified a DMR in the miR-4335 and miR-378 promoters and validated their methylation status by bisulfite sequencing PCR. The hypermethylation of the promoters of miR-4335 and miR-378 in LBF and the resulting silencing of the target genes may result in LBF’s low content in saturated fatty acids and fat deposition capacity. This study provides a solid basis for exploring the epigenetic mechanisms affecting fat deposition and fatty acid composition.

Animal fat is a common part of the human diet, especially in developing countries. However, it is widely accepted that the amount and type of consumed dietary fat has a direct impact on human health. For instance, an excess of dietary fat may significantly contribute to the development of type II diabetes, obesity and other diseases^1^,^2^,^3^,^4^. In addition, the dietary fatty acid composition is also highly related to human health. Recent studies have shown that some saturated fatty acids (SFA), but not mono- or polyunsaturated fatty acids (MUFA and PUFA, respectively), can increase plasma cholesterol levels and the risk of cardiovascular diseases^5^. However, some PUFA and MUFA are known to decrease levels of low-density lipoprotein and cholesterol and also mediate important benefits for human health by stimulating the immune system^6^,^7^,^8^. Additionally, the ratios between PUFA and MUFA and between n-6 PUFA and n-3 PUFA have effects on human health and are used to characterize healthy fats^9^. It is also well known that fatty acid composition directly affects the technological properties of meat products by altering the lipid melting point and fat firmness^10^. The fatty acid composition further affects the tenderness, juiciness and flavor of the meat^11^. Therefore, a better understanding of the mechanisms behind adipose tissue accumulation and fatty acid composition in pigs is of great importance for human health but simultaneously may be helpful in improving pork quality and production efficiency.

In recent decades, the pork industry has focused on increasing the lean meat percentage by decreasing subcutaneous fat content, but the mechanisms behind fat deposition and fatty acid composition regulation are complex physiological processes that, in turn, are regulated by a considerable number of genes. To date, these are not fully understood. Detection of quantitative trait loci by genome-wide association studies based on gene mutations and polymorphisms is most commonly applied to investigate this important economic trait^12^. Zhang *et al*.^13^ identified 865 single nucleotide polymorphisms (SNPs) related to fatty acid metabolic traits in five pig populations^13^. Roger *et al*. (2016)^14^ conducted a genome-wide association study and found that SNPs at the SCD and LEPR loci were the two main loci influencing the intramuscular fat content and fatty acid composition in Duroc pigs^14^. However, it is evident that DNA sequence polymorphism alone does not provide adequate explanations for the mechanisms of fat deposits and fatty acid composition regulation. With the development of next generation sequencing technologies, global changes in the mRNA and microRNA transcriptomes are being analyzed to better understand the epigenetic regulation of fat deposition and fatty acid composition. Xing *et al*. compared the diversity of the mRNA transcriptomes of high (38 mm) and low (12 mm) thickness pig backfat^15^. Mentzel *et al*. reported specific microRNA expression patterns in adipose tissues of lean and obese pigs using RNA sequencing^16^. Moreover, DNA methylation, a type of heritable modification affecting gene expression without changing the DNA sequence itself, is another epigenetic key factor in fatty acid metabolism^17^. Li *et al*. conducted a genome-wide analysis of DNA methylation differences existing between different layers of porcine backfat tissues and found genes related to cytokine release to be hypermethylated in superficial backfat layers^18^. Grundberg *et al*. compared global DNA methylation patterns between adipose tissues from twins and detected disease-associated variants in distal regulatory elements^19^. There are many studies reporting differences regarding the adipose methylome of distinct adipose tissues and individuals, and most of these only demonstrate possible relationships with human diseases^20^,^21^,^22^,^23^,^24^. Nevertheless, we do not fully understand how DNA methylation affects fat deposition and fatty acid metabolism.

Here, we used two well-defined pig breeds displaying distinct fat properties (LP, a leaner western breed, and RP, a fatty breed indigenous to China), collected the subcutaneous fat and performed a genome-wide analysis of DNA methylation differences between their adipose tissues. We identified their DNA methylation landscapes and differentially methylated regions (DMRs). To identify the effect of DMRs on lipid metabolism, we then performed a functional enrichment analysis for genes exhibiting DMRs that were known to affect fat deposition and fatty acid synthesis. The results of this study may serve as a valuable basis for further research regarding healthy human diets and the improvement of pork quality and production efficiency.

## Materials and Methods

### Ethics Statement

All research involving animals and tissue sampling were carried out in accordance with Guidelines for the Regulations for the Administration of Affairs Concerning Experimental Animals (Ministry of Science and Technology, China, revised in June 2004) and approved by the Institutional Animal Care and Use Committee in College of Animal Science and Technology, Sichuan Agricultural University, Sichuan, China under permit No. DKY- DKY-2014-18.

### Animal treatment and tissue collection

Nine female Landrace pigs and nine female Rongchang pigs were used in this study. All pigs were housed in the same environment and slaughtered at 210 days of age. In preparation for slaughtering, food was withdrawn from the animals for 24 hours. However, they did have free access to water during this time. The pigs were electrically stunned, exsanguinated, scalded and rinsed. Landrace pig backfat (LBF) and Rongchang pig backfat (RBF), located between the third and fourth last rib, were obtained immediately, rapidly frozen in liquid nitrogen and stored at −80 °C until RNA and DNA extraction. Similarly obtained samples from the region between the fourth and fifth last rib were used for histologic and fatty acid composition analysis.

### Measurement of body density, adipocyte volume and fatty acid composition

Body density negatively correlates with fat percentage and may be utilized to assess the porcine productive type. We calculated the pigs’ body volume on the basis of the anthropometric parameters radius of abdomen (A) and neck (N) and body length (BL), according to the following formula:


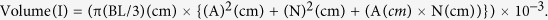


To calculate the animals’ body density, the following, previously used^23^, formula was applied:


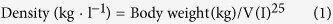


All adipose samples were fixed in a 10% formalin solution and made into paraffin sections with hematoxylin and eosin staining. A TE2000 fluorescence microscope (Nikon, Melville, NY) and Image Pro-Plus 7.0 software (Media-Cybernetics, Bethesda, MD) were used to calculate the diameter of adipocytes. The mean adipocyte volume (V) was obtained by applying the following formula:


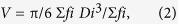


where *Di* is the cells’ mean diameter and *fi* denotes the absolute number of cells with the mean diameter *Di*. The fatty acid composition was analyzed with a GC-^14^C gas chromatograph (Shimadzu, Kyoto, Japan) according to a previously published method^26^.

### Methylated DNA immunoprecipitation sequencing

We randomly selected three Landrace pigs and three Rongchang pigs as biological replicates. Their respective adipose tissues were used to construct MeDIP DNA libraries. Briefly, DNA (5 μg) extracted with the DNeasy Blood & Tissue Kit (Qiagen, CA, USA) was sonicated with a Bioruptor sonicator (Diagenode, city, NJ, USA) to produce approximately 100–500 bp fragments. Subsequently, libraries were constructed using a Paired-End DNA Sample Prep kit (Illumina, CA, USA) according to the manufacturer’s instructions. The fragments were ligated with adaptors and immunoprecipitated using monoclonal anti-methylcytidine antibodies (Diagenode, Denville, NJ). This procedure enabled us to enrich our samples for methylated DNA, which was subsequently purified (DNA Clean & Concentrator-5 columns, Zymo CA, USA) and amplified by adaptor-mediated PCR. The MeDIP libraries were then subjected to paired-end sequencing using an Illumina HiSeq 2000 (Illumina, CA, USA) and a 50 bp read length. Raw sequencing data were processed through the Illumina base-calling pipeline. Methylated DNA immunoprecipitation sequencing (MeDIP-seq) data have been deposited in the NCBI Gene Expression Omnibus under the GEO accession number GSE80096.

### Analysis of MeDIP-seq data and identification of DMRs

After discarding low quality reads that contained more than 5 ‘N’s or low quality values (Phred score < 5) for more than 50% of the sequence, MeDIP-seq data were aligned to the porcine reference genome (Sus scrofa 10.2), allowing for up to four mismatches per read and using SOAP2 software (Version 2.21)^27^. Reads mapping to multiple genomic locations were regarded as duplicates and considered as one read. To avoid stochastic sampling drift, we filtered out CpG sites that were covered by less than a 10 read depth. DMRs were identified as previously published^21^. Normality and homoscedasticity of the read depth at each CpG in LBF and RBF were tested using Bartlett’s test (passing if *P* > 0.05, failing if *P* < 0.05). Subsequently, a parametric (passing Bartlett’s test) or non-parametric test (failing Bartlett’s test) was used to select highly variable CpGs (*P* < 0.01) as seed sites for possible DMR. Then, a 3′ downstream region adjacent to the CpG was incorporated into the seed CpGs (up to 200 bp), and the new seed CpGs were repeatedly joined to the next CpG until appearing as a low-variance CpG (*P* > 0.01), which was allowed to be located up to 2 kb upstream from the seed CpG. If significant different read depths (*P* < 0.01) were demonstrated for five or more CpGs in a genomic region, it was considered to be differentially methylated in Landrace and Rongchang pigs. *P* values for DMR were corrected using the Benjamini-Hochberg method (FDR < 0.01, 1,000 permutations). All DMRs were annotated into 24 genomic elements of porcine genomic regions according to our group’s previous study^28^.

### Functional enrichment analysis

The Database for Annotation, Visualization and Integrated Discovery (DAVID) web server (http://david.abcc.ncifcrf.gov/) was used to perform functional enrichment analyses. First, genes with DMR in promoters were converted to human orthologous genes. Second, these orthologous genes were submitted to the DAVID web server for functional enrichment analyses of Gene Ontology (GO) and pathways. All lists were submitted to DAVID and scanned for significant overrepresentations of GO biological process (GO-BP), molecular function (GO-MF) and cellular component (GO-CC) terminologies, as well as for their KEGG-pathway category. Only Benjamini-corrected values of *P *< 0.05 were considered to be significant.

### Bisulfite sequencing PCR

Methylation Primer Express Software V1.0 was used to design bisulfite sequencing PCR (BSP) primers, which are provided in Table S1. DNA was isolated using the DNeasy Blood & Tissue Kit (Qiagen, CA, USA) according to the manufacturer’s protocol. We acquired bisulfite converted DNA using the EZ DNA Methylation-Gold Kit (Zymo Research, Irvine, CA, USA) following the manufacturer’s protocols. PCR was carried out with the ZymoTaq PreMix (Zymo Research, CA, USA) according to the manufacturer’s specifications. The PCR products were subsequently purified using the DNA Clean & Concentrator - 25 Kit (Zymo Research). Subsequently, the PCR products were cloned into a TA vector (Invitrogen, Carlsbad, CA, USA). Ten effective subclones were selected for each gene, and successful cloning was subsequently confirmed by analyzing the sequence data (BiQ Analyzer V2.0) obtained with an ABI 3730 DNA sequencer (Applied Biosystems, Foster City, CA)^29^.

### Quantitative RT-PCR

Quantitative RT-PCR (Q-PCR) was used to measure mRNA and microRNA (miRNA) expression levels (primers shown in Supplementary Table S1). Total RNA was extracted from adipose tissues using TRIzol reagent (Invitrogen) and further purified with RNeasy columns (Qiagen) according to the manufacturer’s protocol. Reverse transcription of mRNA and miRNA was performed using the PrimeScript RT Master Mix kit and the PrimeScript^™^ miRNA RT-PCR Kit, respectively (both obtained from TaKaRa, Dalian, China) following the manufacturer’s recommendations. Quantitative PCR was performed using the SYBR Green Real-time PCR Master Mix (TaKaRa) on a CF96 Real-Time PCR Detection System (Bio-Rad, Hercules, California, USA). The *ACTB*, *TBP* and *TOP2B* genes were simultaneously used as internal controls for mRNA normalization. Expression levels of U6 served as endogenous controls for miRNA expression and were utilized to normalize the corresponding data. The 2^−ΔΔCt^ method was used to determine the relative mRNA and miRNA abundance^30^.

## Results and Discussion

### Differences in phenotypic traits between LBF and RBF

**Figure 1:**
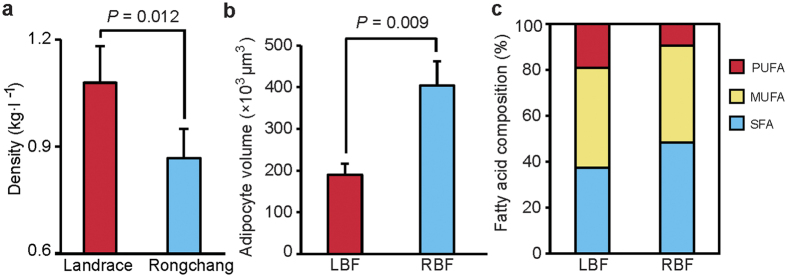
Phenotypic differences between Landrace backfat (LBF) and Rongchang pig backfat (RBF). (**a**) Body density of Landrace and Rongchang pigs; Student’s paired t-test (n = 9). Values are means ± SD. (**b**) Adipocyte volume measured in LBF and RBF; Student’s paired t-test (n = 9). Values are means ± SD. (**c**) Fatty acid composition of LBF and RBF. SFA, MUFA and PUFA are saturated, monounsaturated, and polyunsaturated fatty acids, respectively. Student’s paired t-test (n = 3, tested by absolute content of PUFA, MUFA and SFA, *P*_PUFA_ = 0.006, *P*_MUFA_ = 0.77, *P*_SFA_ = 0.035).

Because of different consumption habits, in Europe, the Landrace breed has been genetically selected for more than 100 years to reduce fat content, whereas the Chinese Rongchang pig has been selected for extreme adiposis. To confirm the suitability of these two breeds as a good model for our study, we measured their respective body densities and adipocyte volumes. As shown in Fig. 1a,b, the Rongchang pig exhibited a lower body density than the Landrace pig (*P* = 0.012), and the adipocyte volume of RBF was almost twice as high as that of LBF (*P* = 0.009). These results suggest that Landrace and Rongchang are typically lean and obese breeds, respectively. In addition, the LBF and RBF tissues exhibited distinct fatty acid compositions in regards to their content of SFA and PUFA (Fig. 1c and Supplementary Table S2). For instance, LBF demonstrated a higher content of C18:2n-6 and C20:3n-3 (16.04% and 1.37%, respectively) than RBF (6.79% and 0.81%, respectively) but lower amounts of C16:0 and C18:0 (22.31% and 13.30%, respectively) when compared with RBF (28.11% and 18.26%, respectively). These results agree with those reported in a previous study that found that fatter pigs exhibited greater proportions of SFA but a lower content of PUFA than the leaner pigs^31^. These results were also in accordance with PUFA composition in humans with different body mass indexes (BMIs)^32^,^33^. Other scientists have found that contrary to MUFA or PUFA intake, over-consumption of SFA may increase the risk of cardiovascular diseases^7^. Therefore, the recommended ratio of PUFA to SFA should be >0.4^34^. However, we found this ratio to be higher in LBF (0.51) than in RBF (0.19) and thus suggest that LBF may be more beneficial to human health than RBF. According to the phenotypic differences between both breeds, their rates of fat deposition and mobilization and their synthesis of fatty acids differ considerably^35^. This also seems to imply that DNA methylation may be one of the important molecular regulation mechanisms involved in this biological pathway.

### MeDIP-seq data and characterization of DMR

**Figure 2:**
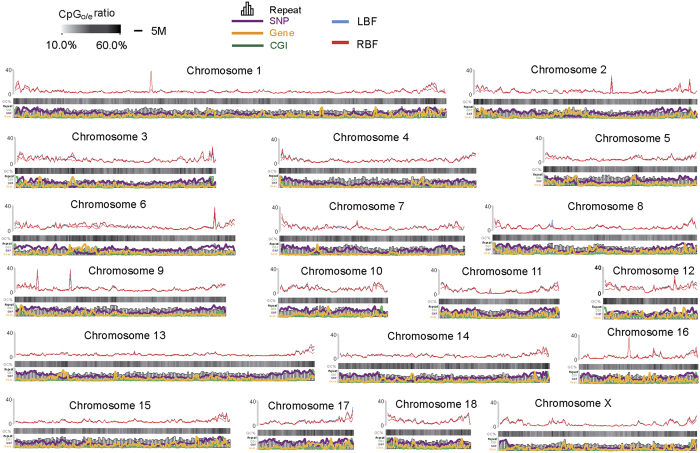
Chromosomal profiles of LBF and RBF methylomes. The distribution of DNA methylation levels throughout the pig genome was analyzed. To compare DNA methylation ra stes among samples, read depth was normalized to the overall average amount of reads in each group, and then a 1 Mb sliding window was used to smooth the distribution. CpG_o/e_ ratio, SNP density, gene, repeat and CGI were all calculated based on this 1 Mb sliding window.

To explore the global differences in DNA methylation between LBF and RBF, we generated 38.27 gigabases (Gb) of MeDIP-seq data from six samples, representing approximately 15 times the size of the pig genome. Approximately 88.89% of all reads could be aligned to the pig reference genome, and approximately 76.68% of the reads corresponded to a unique genomic location (Supplementary Table S3). Subsequently, we used statistics to measure the methylation rate differences in LBF and RBF tissues and defined the DMR. One Mb sliding windows were calculated to obtain an overview of DNA methylation across the porcine genome (Fig. 2). The correlation between methylation levels and genomic features were also assessed, and we found that the methylation levels across chromosomes negatively correlated with the chromosomal length (Pearson’s *r* = −0.728, *P* = 4.11 × 10^−4^) but correlated positively with GC content (*r* = 0.800, *P* = 3.94 × 10^−5^), the observed and expected numbers of CpG sites (CpG_o/e_) (*r* = 0.907, *P* = 7.41 × 10^−8^) (Supplementary Fig. S1). Similar results were reported in a previous study on porcine DNA methylomes^28^.

**Figure 3:**
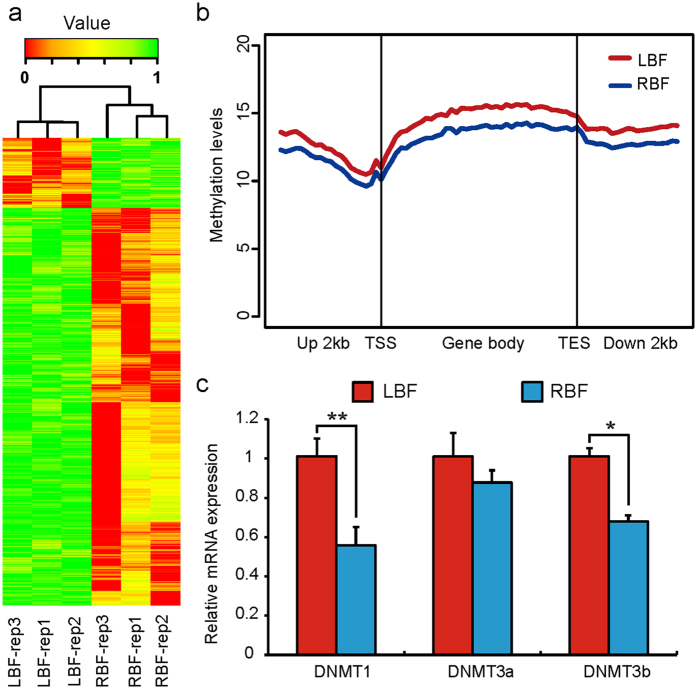
Distribution of MeDIP-Seq reads in the gene region. **(a)** Hierarchical clustering analysis for biological reproducibility. The 0-1 colour code indicated the relative methylation levels (0, lowest methylation level; 1, highest methylation level). **(b)** Distribution of reads around gene bodies. The x-axis indicates the position around gene bodies, and the y-axis indicates the normalized read number. **(c)** Relative mRNA expression levels of DNMT in LBF and RBF. The expression levels were normalized to LBF. (Student’s *t*-test, **P < 0.01, *P < 0.05).

The reproducibility and reliability of MeDIP-seq libraries were also analyzed by DMRs using hierarchical clustering. As shown in Fig. 3a, there was a high uniformity between samples obtained from each of the two breeds (*r *> 0.93). These results confirm the high reproducibility and reliability of those MeDIP-seq libraries used in this study. To achieve a better understanding of the distribution of DNA methylation at the gene level, we further divided the landscape into regions ranging from 2 kb upstream of the TSS of the gene body to 2 kb downstream of the TES (Fig. 3b). LBF exhibited higher methylation levels than RBF in the whole gene region. To determine whether the global reduction in DNA methylation observed in RBF was associated with alterations in DNMTs^36^, we evaluated mRNA expression levels of DNMT1 (the major maintenance methyltransferase), DNMT3a and 3b (*de novo* methylation methyltransferases) in LBF and RBF (Fig. 3c). We found the expression levels of DNMT1 and DNMT 3b in LBF to be significantly higher than in RBF. This result is in accordance with a previous report, which found that the knockout of DNMT1, DNMT3a and DNMT3b did indeed induce a global reduction in DNA methylation^37^. In addition, the number of DMRs distributed among the 5 defined features of canonical gene structures was measured (Table 1 and Supplementary Table S4). LBF exhibited approximately 3–6 times as many hypermethylated genes than RBF across all the 5 gene elements, and the intergenic regions (59%) possessed a maximum number of DMRs higher than other gene regions. It has been suggested that the low capacity of fat deposition of Landrace pigs may be caused by hypermethylated genes that hinder the synthesis of fatty acids.

### Functional enrichment analysis of genes with DMRs

**Figure 4:**
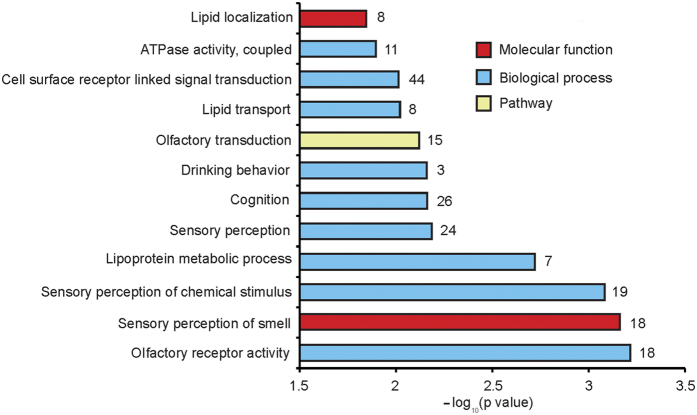
Top twelve GO (Gene Ontology) categories enriched for genes with DMRs in their respective promoters. The number at the right to each bar is the number of genes that significantly enriched in each GO term.

**Figure 5:**
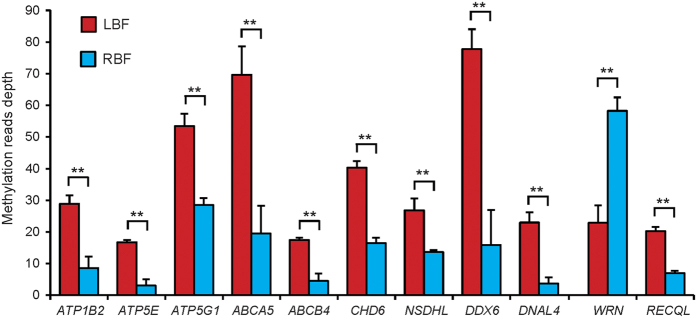
Differential DNA methylation in the promoters of 11 genes involved in ATPase activity. *ATP1B2*: ATPase Na+/K+ transporting subunit beta 2; *ATP5E*: ATP synthase H+ transporting mitochondrial F1 complex epsilon subunit; *ATP5G1*: ATP synthase H+ transporting mitochondrial Fo complex subunit C1 (subunit 9); *ABCA5*: ATP binding cassette subfamily A member 5; *ABCB4*: ATP binding cassette subfamily B member 4; *CHD6*: chromodomain helicase DNA binding protein 6; *NSDHL*: NAD(P) dependent steroid dehydrogenase-like; *DDX6*: DEAD-box helicase 6; *DNAL4*: dynein axonemal light chain 4; *WRN*: Werner syndrome RecQ like helicase; *RECQL*: RecQ like helicase. Student’s paired t-test (n = 3). **P < 0.01.

To explore the metabolic function of differentially methylated genes, we performed an enrichment analysis of Gene Ontology (GO) for genes with a DMR in their promoters^38^. We found that the top 12 significantly overrepresented GO terms were related to two main classes of biological functions (Fig. 4 and Supplementary Table S5), *i.e.*, olfactory and sensory activity (encompassing olfactory receptor activity and the sensory perception of smell and olfactory transduction)^39^,^40^ and lipid metabolism (including lipoprotein metabolic processes, lipid transport, lipid localization and ATPase activity). Notably, the most significantly enriched category was olfactory and sensory activity, and differences regarding olfaction and sensory perception most likely trigger distinct dietary habits in Landrace and Rongchang pigs. The Landrace is a highly commercialized breed, which requires a more diverse olfactory gene expression to recognize odors disseminated from a wider range of food types that usually contain certain food additives, such as flavoring agents, to support the pigs’ higher feed intake and pork yields^41^,^42^. However, the Rongchang pig is a semi-grazing breed indigenous to China, which is traditionally fed with simple green forage. Interestingly, we found 16 overrepresented genes to be enriched in the biological processes of lipid transport and localization. The vast majority of those genes showed hypermethylated promoters (93.75%; *i.e.*, PLIN1, BDKRB2, NSDHL, APOL1 and APOL4) in LBF. With regards to PLIN1, Grahn *et al*. found that an augmented expression of this gene could increase the average size of lipid droplets and thus cause the formation of unilocular adipocytes^43^. NSDHL encodes a sterol dehydrogenase or decarboxylase that is involved in the sequential removal of two C-4 methyl groups in post-squalene cholesterol biosynthesis and thus promotes the accumulation of lipid droplets^44^. This suggests that these genes have a higher mRNA expression level in RBF than in LBF, which may result in higher lipid synthesis rates in RBF. This is consistent with our results regarding higher adipocyte volumes in RBF (Fig. 1b). Lipid accumulation is due to an enhanced synthesis of fatty acids, glycerol and triglycerides and is, therefore, dependent on different processes in lipid transport and localization^45^,^46^. In this study, we also found 11 significantly overrepresented genes to be enriched in the category of ATPase activity. As has been the case for genes enriched in lipid transport and localization, promoters of 90.91% of these genes were hypermethylated in LBF (Fig. 5). For instance, this applies to ATP5E and ATP5G1, which play an essential role in ATP synthase biosynthesis and assembly and thus affect ATP synthesis and mitochondrial energy provision^47^,^48^. ABCA5 and ABCB4 proteins bind ATP and use the energy to drive the transport of very long chain fatty acids across the plasma membrane and intracellular membranes^49^,^50^. Therefore, this suggests that Landrace pigs have a lower energy metabolism level, which may result in lower lipid synthesis efficiency when compared with Rongchang pigs.

### The difference in DMRs of genes impacting fatty acid composition

Fatty acid composition in adipose plays an important role in human healthy diet and is closely related to the conversion of SFA to PUFAs by the activity of desaturases and elongases^51^. PUFA biosynthesis depends on several desaturases and elongases, particularly on the Δ5-, Δ6-, Δ8- and Δ9-desaturases. Stearoyl-CoA desaturase (SCD) 1 for instance, a Δ9-desaturase, plays a key role in transforming SFA to MUFA. It is involved in the biosynthesis of oleate and palmitoleate^52^. In contrast, SCD5, a newly identified isoform of the SCD family, is a Δ8-desaturase^53^. FADS2 and FADS1 encode Δ6 and Δ5 desaturases, respectively, which catalyze the initial and rate-limiting desaturation of linoleic acid to γ-linolenic acid and of α-linolenic acid to stearidonic acid^54^,^55^. In this study, we found higher shares of PUFA in LBF than in RBF (19.07% and 9.43%, respectively, *P* < 0.01). It is tempting to speculate that Landrace pigs are able to convert SFA to MUFA with a higher efficiency than Rongchang pigs. However, no DMR has been detected in the promoters of the four genes related to PUFA biosynthesis. It is suggested that there are other mechanisms involved in the regulation of fatty acid composition that may explain the existing differences in adipose tissue properties, such as post-transcriptional regulation.

**Figure 6:**
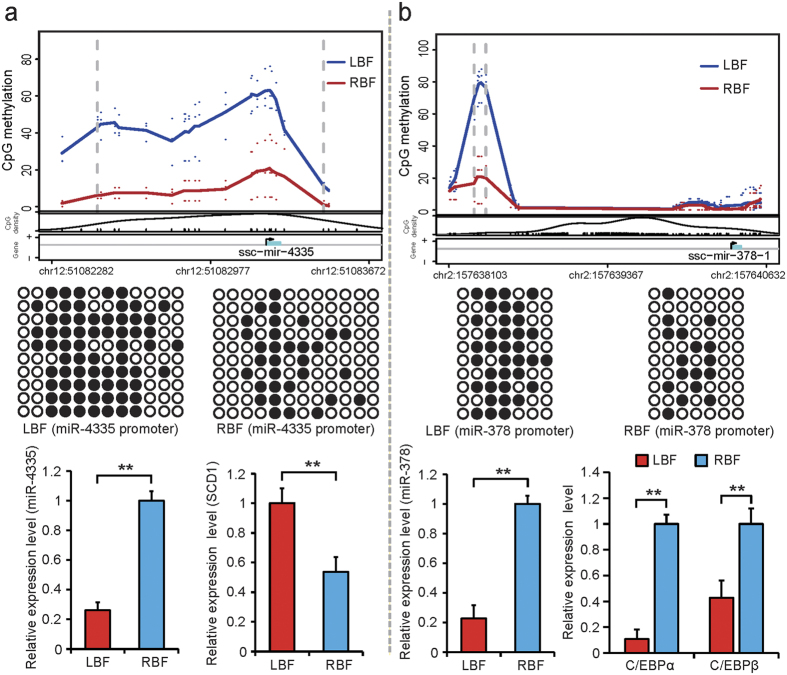
Differentially methylated promoters of miRNA involved in fatty acid metabolism. (**a**) DMR in miR-4335 promoter. (**b**) DMR in miR-378 promoter. For upper panels, each point represents the methylation level (MeDIP-seq read depth) of a sample at CpG site. The curves depict average percentages of all samples. The two vertical dashed lines mark the boundaries of the identified DMR. Middle panels show the validation of CpG methylation by BSP. Ten subclones were selected for the BSP analysis. Solid circles represent methylated CpG sites, and open circles represent unmethylated CpG sites. Lower panels display relative expression levels of genes in LBF and RBF. Gene expression levels were detected by Q-PCR and normalized to the expression levels in high one. Student’s paired t-test (n = 3). **P < 0.01.

miRNAs are small non-coding RNA that can post-transcriptionally regulate gene expression due to partial complementarity to the 3′ untranslated region of their target genes^56^. Previous studies have reported that hypermethylated promoters of genes encoding for miRNA could significantly decrease the corresponding miRNA expression levels^57^,^58^. Interestingly, we found the promoter of miR-4335 to be hypermethylated in LBF. This finding was confirmed in a BSP, and the hypermethylated promoter of miR-4335 in LBF caused a significant decrease in miR-4335 expression (Fig. 6a, *P* < 0.01). Subsequently, we used two software programs (TargetScan and PicTar) to predict the target genes of miR-4335 and found SCD1 to be a well-characterized target gene with full complementarity to the seed sequence of miR-4335 (Supplementary Fig. S2). We then assessed the expression levels of SCD1 and found the mRNA expression level of SCD1 in LBF to be higher than in RBF. SCD1 is well known as a Δ9-desaturase and can catalyze the conversion of SFA to MUFA^52^, which may be a key factor leading to the lower SFA percentages in LBF (37.39% and 48.42% in LBF and RBF, respectively). Furthermore, we also found that the promoter of miR-378 displayed higher methylation levels in LBF than in RBF, which was also confirmed by BSP (Fig. 4b). Gerin *et al*. found that miR-378 could increase the size of lipid droplets and promote the incorporation of acetate into triacylglycerol by specifically increasing the transcription dependent on C/EBPα and C/EBPβ^59^, which is in accordance with our finding that the hypermethylated promoter of miR-378 caused a reduced expression of miR-378 and consequently a decrease in the expression of C/EBPα and C/EBPβ (Fig. 6b). We propose this as an important reason for the higher adipocyte volume and fat deposition capacity measured in Rongchang pigs.

## Conclusion

In summary, we present epigenetic evidence of functionally relevant methylation differences between obese and lean pigs’ backfat tissues. We found that the genes regulated by differentially methylated promoters were mainly involved in olfactory and sensory activity, lipid metabolism and ATPase activity, which reflects the fact that these two breeds have different dietary habits, fatty acid compositions and energy metabolism levels. Moreover, the DMR in the promoter of miR-4335 may also influence the fatty acid composition in backfat by targeting SCD1 (Δ9-desaturase). The hypermethylated promoter of miR-378 may decrease the expression of C/EBPα and C/EBPβ in LBF and thus cause higher adipocyte volumes and an enhanced capacity for fat deposition.

## Additional Information

**Accession codes:** The high-throughput sequencing data have been deposited in NCBI’s Gene Expression Omnibus under GEO Series accession numbers GSE80096.

**How to cite this article**: Zhang, S. *et al*. DNA methylation landscape of fat deposits and fatty acid composition in obese and lean pigs. *Sci. Rep.* **6**, 35063; doi: 10.1038/srep35063 (2016).

## Supplementary Material

Supplementary Table S4_S5

Supplementary Figures and Tables

## Figures and Tables

**Table 1:** Number of genes showing differentially methylated genes in DMRs.

LBF vs. RBF	Differentially methylated gene				
	Promoter	Exons	Introns	Downstream 2 kb	Intergenic
Hyper-methylated	406	223	4489	322	7962
Hypo-methylated	77	78	809	58	1338
