# Supplementary material for: DNA methylation landscape of fat deposits and fatty acid composition in obese and lean pigs
Source: Sci Rep. 2016 Oct 10;6:35063. doi: 10.1038/srep35063 (PMC5056348; doi:10.1038/srep35063)
Supplement: Supplementary Figures and Tables [file srep35063-s2.pdf]

Supplementary Information

DNA methylation landscape of fat deposit and fatty acid composition variation in obese and lean pigs

Shunhua Zhang, Linyuan Shen, Yudong Xia, Qiong Yang, Xuewei Li, Guoqing Tang, Yanzhi Jiang, Jinyong Wang, Mingzhou Li & Li Zhu

On-line Supplementary Data

Supplementary Figures:

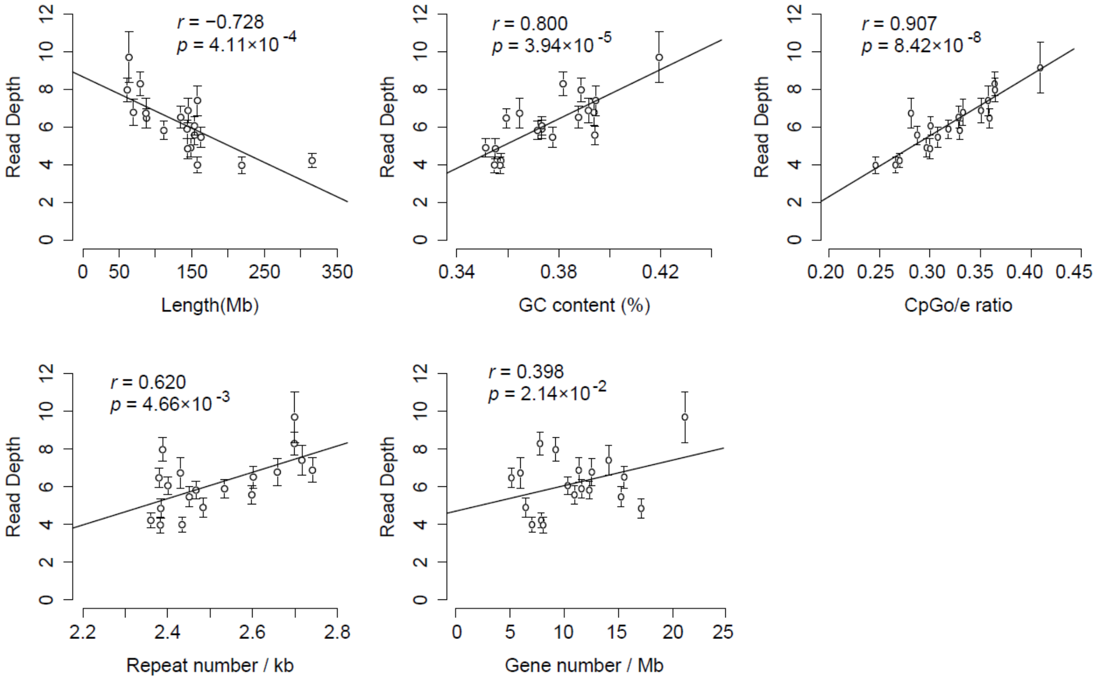

Supplementary Figure S1. Pearson's correlation between DNA methylation levels and chromosomal features.

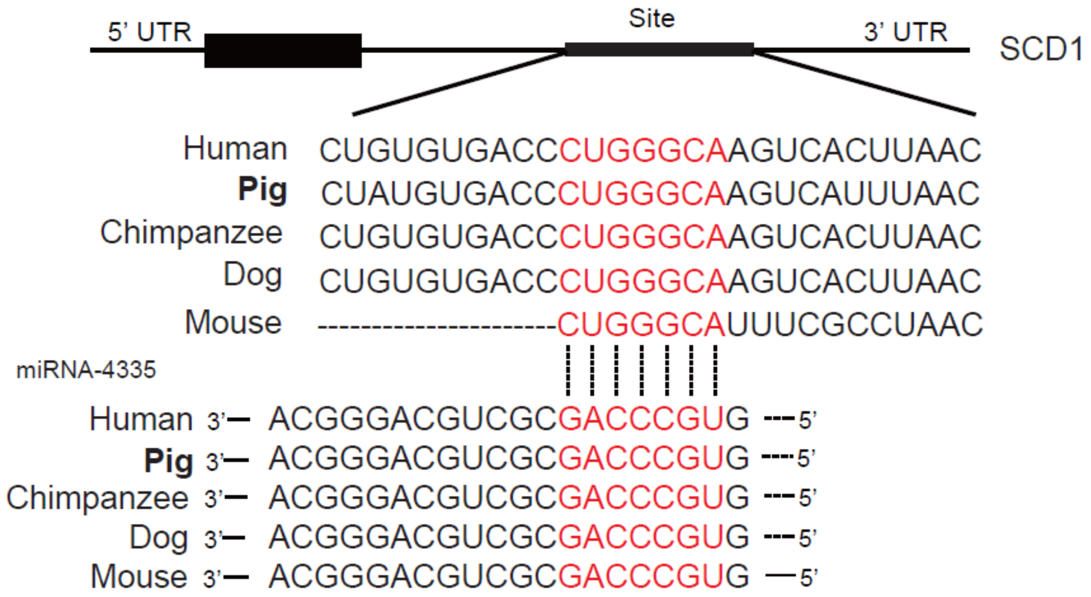

Supplementary Figure S2. miR-4335 targets the 3'-UTR of SCD1. Sequence alignment of miR-4335 with 3'-UTR of human, pig, chimpanzee, dog and mouse SCD1 mRNA. Binding site and seed region of miR-4335 are indicated in red.

Supplementary Table S1: Information on primers used to perform Q-PCR and BSP.

Gene symbol	Forword primer	Reverse primer
C/EBPα	GGCGGCATCTGCGAACACGA	GCTGAAACAGGTCGGCCAGGAA
C/EBPβ	CCTGTCCACATCCTCGTCGTCC	CGCTGTGCTTATCCACGGTCTTCT
SCD1	TGAGGGCTTCCACAACTAC	TGGCAGCCTTGGATACTT
DNMT1	TGGCGGGACCTACCAAACA	ACTTCCACGCAGGAGCAGA
DNMT3a	AAGAATGCCACCAAATCAGCC	AGAACTTGCCGTCTCCGAACCA
DNMT3b	AGGTCTCCAGCCTCCTAAGTT	GTGTCTGAGCCATCTCCATCC
miR-4335	GUGCCCAGCGCUGCAGGGCA	Uni-miR qPCR Primer, included in kit
miR-378	ACUGGACUUGGAGUCAGAAGGC	Uni-miR qPCR Primer, included in kit
BSP-4335	GAGAGAGAGGTTTTAGATTGATGG	CCACTTCCTACATACAAAAAAATC
BSP-378	TAATTTGGGAAATGTAATTGAGAG	ACCTCTCTCCCCAACTATAAAATA
U6 snRNA*	TTATGGGTCCTAGCCTGAC	CACTATTGCGGGTCTGC
ACTB*	TCTGGCACCACACCTTCT	TGATCTGGGTCATCTTCTCAC
TBP*	GATGGACGTTCGGTTTAGG	AGCAGCACAGTACGAGCAA
TOP2B*	AACTGGATGATGCTAATGATGCT	TGGAAAAACTCCGTATCTGTCTC

* denotes the endogenous control genes. ACTB, TBP and TOP2B were simultaneously used as internal gene for mRNA normalization, but U6 was used as endogenous controls for microRNA. BSP-4335 and BSP-378 primer was designed for bisulfite sequencing PCR.

Supplementary Table S2: The fatty acid composition of LBF and RBF

Items	LBF	RBF	P value
**C10:0**	0.04	0.04	NS
**C12:0**	0.05	0.06	NS
**C14:0**	1.00	1.34	**
**C16:0**	22.31	28.11	**
**C16:1**	1.74	1.63	NS
**C17:0**	0.36	0.20	**
**C17:1**	0.25	0.12	**
**C18:0**	13.30	18.26	**
**C18:2n-6**	16.04	6.79	**
**C18:3n-3**	0.76	1.24	**
**C18:3n-6**	0.04	0.01	**
**C18:n-9**	41.38	40.24	NS
**C20:0**	0.34	0.40	*
**C20:2**	0.80	0.54	**
**C20:3n-3**	1.37	0.81	**
**C20-1**	0.15	0.13	NS
**C22n-9**	0.02	0.02	NS
**DHA**	0.05	0.03	*
**EPA**	0.02	0.02	NS
**SFA**	37.39	48.42	**
**MUFA**	43.54	42.15	NS
**PUFA**	19.07	9.43	**

DHA: Docosahexaenoic acid (22:6 n-3); EPA: Eicosapentaenoic acid (20:5 n-3); SFA, MUFA and PUFA are saturated, monounsaturated, and polyunsaturated fatty acids, respectively. Student's paired t-test (n = 9). ***P* < 0.01, **P* < 0.05, NS: no significant difference (*P* > 0.05).

Supplementary Table S3: Summary of MeDIP-seq data production.

Tissue symbol	Biological replicate	Number of raw reads	Raw reads (Gb)	Number of clean reads	Clean reads (Gb)	Number of aligned reads	% aligned reads	Number of unique reads	% of unique reads
LBF	**1**	143,011,442	7.01	137,770,430	6.89	122,064,346	88.60%	93,211,480	76.36%
	**2**	131,080,312	6.42	124,543,412	6.23	111,321,020	89.38%	85,807,761	77.08%
	**3**	142,136,092	6.96	132,197,744	6.61	118,842,650	89.90%	93,061,223	78.31%
RBF	**1**	114,984,412	5.63	110,322,514	5.52	95,528,268	86.59%	71,905,416	75.27%
	**2**	110,543,488	5.42	106,464,046	5.32	94,274,142	88.55%	70,451,004	74.73%
	**3**	126,839,390	6.22	121,115,662	6.06	109,004,233	90.00%	84,795,274	77.79%
**Total**		**781,057,914**	**38.27**	**732,413,808**	**36.63**	**651034659**	**88.89%**	**499232158**	**76.68%**

The low-quality reads were filtered from the raw reads and clean reads were used in further analysis. “% aligned” is percent of clean reads aligned on the pig reference genome (version 10.2). “% unique” is percent of reads uniquely aligned over all the aligned reads. The reads with same mapping locations in each sample were taken as potentially duplicated clones created by PCR amplification during sequencing library construction, and hence were removed in the analysis.
